# A Memoir of the Life, Character, and Writings of the Late Prof. John Harrison, M. D.

**Published:** 1850-02

**Authors:** 


### ECLECTIC DEPARTMENT,

### AND SPIRIT OF THE MEDICAL PERIODICAL PRESS.

*A Memoir of the Life, Character, and Writings of the late* Prof. John Harrison, M. D., read before the Physico-Medical Society of New Orleans, August 11th, 1849, by James Jones, M. D., A. M., Professor of the Theory and Practice of Medicine in the Medical Department of the University of Louisiana. Published by order of the Society.

Dr. John Harrison, professor of Physiology and Pathology in the Medical Department of the University of Louisiana, was born at the Navy yard in Washington City, on the 30th of August, 1808, baptized a few days afterwards, and named after his maternal grandfather, John Hoffman Harrison. His parents were of old and respectable families in the State of Maryland—his father, a surgeon of good standing in the Navy, from whom it is probable he imbibed a predilection for the medical profession. He was not permitted long to enjoy the society and care of his mother and father, the former dying when he had reached the age of ten, and the latter several years after, leaving him under the charge of kind relations in Georgetown, D. C., by whom he was placed at the grammar school of the celebrated Dr. Carnahan. At this institution, he received an excellent classical education, one, indeed, equal to that of our most respectable colleges. He was always known as an intelligent boy, although no evidence of his capacity or peculiar tastes were exhibited until his fifteenth year, when he entered on the study of algebra; the delight in which he displayed, was the commencement of those tastes for abstract subjects, which were so remarkably developed in after life.

Small in stature and of a delicate frame, he was withal capable of enduring great fatigue, and extremely fond of swimming, hunting and other active and manly amusements. He was of a quick and indomitable spirit, which made him respected by all invaders of the rights of others, and gave to his maturity that fearless and chivalrous character, for which he was to all of us so well known. An orphan, and without companions at home of his own age, he found among his school-mates a grateful return of the sympathies which nature had made active in his heart, and already formed, in early youth, some of those strong and devoted friendships which characterized every other stage of his existence. Left without parental control, responsible to none for the minor duties of a boy's life, he acquired an early reliance on his own resources, an independence of character, which, in him, was most fortunately blended with a native sense of justice and truth.

His classical education completed, our friend was removed to the domicile of his grandfather, at Fredericktown, in Maryland. Here he made choice of the medical profession, and became a student of his relation, Dr. Baltzell, an experienced and intelligent physician, with whom he remained until his graduation in the University of Maryland, in March, 1831. He certainly did not appear to place a very high estimate on the advantages offered by the celebrated school just mentioned, and left it with no exalted opinion of the professors. Let us except, however, professor N. R. Smith, and the lamented Debutts, whose eloquent and brilliant course of chemical instruction doubtless increased our friend's early taste for that science, which he ever afterward retained. As much, if not more, of his attention had been devoted to the collateral sciences, and to literature, as to medicine, and he had already established, among his friends in Frederick, a reputation for vigor and acuteness of intellect, and for a cultivated taste in philosophical and abstract speculations, rarely attained by one of his age.

In 1831, Dr. Harrison had concluded to establish himself in the city of New Orleans, to which he was doubtless inclined by the success and countenance of his maternal uncle, then, as now, an eminent member of the Louisiana Bar. He arrived in the month of November, and soon found himself surrounded by friendly and kindred spirits, some of whom were to influence his future character and prospects in society. Thrmas Watkins Leigh, and Jesse Burton Harrison, were among these, the most prominent. To the former, he owed much of his increased knowledge and refined taste in every department of Fnglish literature; by the latter, he was apparently first stimulated to undertake the study of German, Italian, and French writers, in whose works he has been for some years so remarkably proficient. Devoted so much to extra professional pursuits, extremely youthful in appearance, and apparently not anxious to engage extensively in the practice of medicine, his business for several years was rather limited.

In September, 1832, he made his first effort in this Society, having volunteered to read an article on the propagation of cholera, in which he sustained the agency of contagion. Although equal to the importance of this subject, this essay scarcely sustained the reputation he enjoyed at that time, particularly as regarded the style, which was florid, imaginative and very different from the severe models, on which all of his later productions have been so successfully formed. Indeed, I may here add in connection with this statement, that our friend not only possessed a cultivated taste for English verse, but was himself poetically inclined, and has left behind him a number of effusions written, probably, between 1831–33, during the first two years of his residence in this city.

In September, 1832, he had a severe attack of yellow fever, after which, passing with some professional credit through the cholera of 1832–33, and the yellow fever of the last season, he was elected in October to the important post of resident physician and surgeon in the Charity hospital. This office was in those days one of the most responsible a young man could hold, all of the surgical and much of the medical practice being under his control. It must be confessed that Dr. Harrison did not derive the scientific knowledge, and certainly not the extensive practice, which had accrued to other occupants of that post, and was naturally to be expected from one of his abilities. The air of the house was not congenial to his health; his tastes at that time were more literary than medical, and he himself has mentioned that here he pursued his studies in the modern languages, and made at his leisure hours those numerous voluminous excerpta that we find among his manuscripts. As to the increase of his professional business, I have no doubt that he was actuated by those old-fashioned notions of propriety, which forbade public officers, who were liberally salaried for the use of their time and talents, from disposing, to their own advantage, of that over which they were supposed to have no right of control.

His position having made him necessary to the gentlemen who first constituted the Faculty of the Medical College of Louisiana, he received the appointment of Demonstrator and adjunt professor of Anatomy. Enjoying in the Charity Hospital unlimited opportunities for the cultivation of practical anatomy, he acquired therein considerable proficiency, and laid a good basis for undertaking the duties of the chair of Physiology and Pathology, in his election to which the succeeding year of 1835, the other professors did proper justice to the most able and talented of their faculty. In 1836 he resigned his office in the hospital in favor of the assistant surgeon, and was himself elected one of the visiting physicians.

Never was a subject better adapted to individual tastes and abilities than physiology to Harrison. He entered on the duties of his new chair with enthusiasm, and in the course of a few years had read, examined and mastered every thing known in the science, besides going through various series of experimental inquiry, of which last, unfortunately, no notes, to our knowledge, remain. For thirteen successive years, with the exception of two, in which he was obliged to take the chair of Anatomy, did he deliver one of the most elaborate, learned and scientific courses on Physiology ever heard by any audience, establishing for the professor the enviable reputation of a teacher of the highest rank, and adding greatly to the character and attendance of the institution with which he was associated. The only objection that I have ever heard urged against this course of instruction has been, that it was of too elevated and learned a standard for the majority of those in attendance, and required more knowledge of the elementary sciences, and practiced habits of mental discipline, than they could be reasonably expected to prssess. Admitting the fact, we doubt the justness of the criticism, and consider it the highest compliment that such a course could receive. In truth, amongst the educated and intelligent portion of the class, this course was more highly appreciated than any other in the college.

His reputation and popularity advanced simultaneously with the increase of the class, the beautiful and philosophical discourse which he pronounced at the opening of the session of 1847–48 being published at their request, as an evidence of their admiration of the production, and their esteem for the author.

It was reasonable to presume that one gifted with Dr. Harrison's order of abilities, and so accustomed to the use of his pen, would not be tardy in finding an occasion for presenting his views on subjects connected with his favorite science in some permanent form. A suggestion in an extract from Dr. Stokes' lectures in the American Journal of Medical Sciences for August, 1835, was, according to his own announcement, the origin of his first effort as an author. In this lecture, Dr. Stokes had applied the doctrine of isomerism to the explanation of certain obscure phenomena of lesions of innervation; upon a mature study of which Dr. Harrison conceived the idea of writing his Essay towards a correct theory of the nervous system. This work was a long time in preparation, remained for a considerable period in the hands of the publishers, who entertained some fears as to the success of so abstract and speculative a production, and finally came forth in 1844. Whatever unfavorable surmises may have prevailed as to the prospects of a treatise of this nature, they were rapidly dispelled by the general approbation with which it was greeted by the profession, and the flattering comments of our most respectable American and foreign scientific journals. The literary and scientific reputation of our friend was not only firmly established, but this little work, to the publication of which he anticipated a heavy pecuniary contribution, became, to his own surprise, a source of some revenue.

In 1845, Dr. Harrison entered upon a new field, by the association of himself and Dr. Carpenter with the original editors of the New Orleans Medical and surgical Journal. They both contributed largely to this respectable periodical. To the former, it was indebted for several valuable papers, which will be mentioned particularly hereafter, and for various able reviews, and admirable selections and translations for the miscellaneous department, which added greatly to its circulation and general reputation. So justly were his labors in this department of Medical literature appreciated by his colleagues in the University, that on expressing a desire to retire from his editorial duties in January, 1848, they offered a voluntary contribution of $500 per annum as an expression of their estimation of his services, the publisher offering an equal amount. Our friend remained single until the close of 1842, when he was united to a relative who possessed every requisite for the promotion of his domestic happiness, and in the course of a few years, he became the father of two fine boys, who survive.

Arrived at a period of existence when the intellectual powers are in their full maturity and vigor; happy in his little family; enjoying a select and respectable practice; surrounded by admiring and devoted friends, and in the zenith of his reputation, our lamented associate perceived the first approaches of the disease whose slow and fatal progress was to darken the prospect of a bright future, and ere long to draw the curtain forever between him and all that he valued in life.

In March, 1847, he had a hæmoptisis. We all appreciate too well the gloomy advent of that unhappy messenger. His thoracic symptoms were otherwise so slight that he and his friends flattered themselves that the hemorrhage came entirely from the larynx and posterior nares. He suffered very little with cough, regained much of his ordinary appearance, and having been lately dyspeptic and weak, determined on visiting the American Medical Convention at Philadelphia, in company with our late lamented colleague, Dr. Wm. M. Carpenter. They were duly appointed delegates from this society and from the Medical Department of the University. Never were medical associations better represented. Eminent in the departments of medicine they professed—distinguished by their knowledge and contributions to the natural sciences; well known as authors and scholars, they everywhere received, from men of education, and from the members of their own profession, the respect and attention to which their merits were entitled. In the organization of the American Medical Association, Dr. Harrison was elected a vice president, and both of them were appointed on the most important standing committees. Never, we repeat, were the medical men of Louisiana better represented than by Harrison and Carpenter. Yet in the prime of manhood, they stood on the verge of that limit set to members of a profession which sacrifices every consideration of life to the objects of its high calling, and carried on their wan fronts the badge of honorable toil in an insalubrious and ungrateful climate, as a sad and eloquent contrast with the happier auspices of professional labor in more genial latitudes, where health and independence are the associates of eminence, where talents and scientific attainment give to their possessors the highest position in educated and liberal communities.

Dr. Harrison, during an absence of several months, enjoyed uninterrupted health, and returned home early in August, much improved in appearance, but no sooner re-entered the atmosphere of the city than he felt again its pernicious influence. He once more withdrew to the society of his family in the country, where he remained at a great sacrifice to his business, until the opening of the collegiate session of 1848–49. Under every disadvantage of ill health and depression of spirits, he discharged the duties of his chair ably and faithfully, and likewise attended to the greater part of his practice. While paying the tribute of this society to the memory of a deceased associate, how can I avoid here a grateful recollection of the constant and kind attentions I experienced in my own person, from him and many others of the profession, during a protracted and dangerous illness in the winter of 1848, in which, doubtless, the many long hours of rest that he sacrificed to the care of another had no little influence in hastening the result of his own disease.

With the spring returned also his hemorrhages, several of which occurred in my presence, constant cough, wasting hectic, and acute pains in the thorax announced the rapid advance of the malady we had so much dreaded. He returned to the city, much altered in appearance, last November, though still buoyed up by those delusions so common in Pthisis pulmonalis, rallied for a brief period, and commenced his lectures, which he continued irregularly for a few weeks. Exerting himself imprudently in going to the dead house of the hospital during the commencement of the cholera in December, he became considerably worse, retired to his chamber, and never attempted to lecture again. It was a painful and distressing illness. Exhausted by cough, hectic and profuse sweats, tortured by pleurisies and violent neuralgic pains, his noble intellect triumphed over every calamity, and reigned pure and unclouded to the last moment. For weeks after his confinement, he pursued his favorite studies at tranquil intervals with all the ardor of the most favored periods of his life, investigated anew in the recent editions and later works of Liebig, Berzelius, Gerhardt, Regnault, Pelouze and Fremy, those formulæ and theories of general and organic chemistry, which constituted the striking and beautiful fentures in his physiolophical system, and revisited in Spinosa, Coleridge, McIntosh, Bacon, Pascal and Humboldt, those ethical and philosophical creations which had been the charm of his intellectual existence. To beguile the wearier hours, he re-read in the volumes of older English poetry, the familiar pages of which so many glowing passages lived in his memory, and often gave language to his own matured and regulated thoughts. Confined to his bed, and incapable of supporting a book, he devised an ingenous mechanical expedient, by which it could be held, and this at length overcoming his declining strength, he had yet a resource in the voice of one who, with the tender devotion exhibited during his long illness, read for hours at his bedside.

Thus closed the life of Dr. John Harrison, in the 41st year of his age, on the morning of March 19th, 1849. His remains were followed to their last resting place by a large concourse of friends, colleagues and pupils, and every tribute of respect rendered to his memory by this society and the Medical Department of the University of Louisiana. We opened our winter campaign sadly, with the obsequies of the lamented Carpenter; it had a no less unhappy termination in the same melancholy rites to Harrison. They were friends and associates in life, did much to promote the cause of science, and to elevate the character of this society, and of the medical profession in the State of Louisiana. Their memories will be honored so long as our institutions and records shall remain to perpetuate them.

We have thus comprised, in this memoir, all that its limits will admit, of our friend's biography, and much on the subject of his character. From what has been already stated of the latter, it will, I trust be made to appear that his claims to distinction in science and literature were of no ordinary character. To eminent abilities, he brought studious and methodical habits. Conversant with all that was known in physiology, he was equally learned in metaphysics, logic, and ethical philosophy. Of all his intellectual gifts, that for analysis and generalization was the most remarkable and most forcibly exemplified in his lectures, writings, and daily conversation. In the pursuit of scientific knowledge, his greatest interest was exhibited in the investigation of systems, doctrines, and general principles. In the fundamental laws of chemistry, zoology, physics, and geology, none could be more accurately or critically informed, while to the empirical and practical details so interesting to most inquirers, he was comparatively inattentive.

In English literature, there was probably little of consequence with which he was not perfectly familiar. The same may be averred of the French, German, and Italian. He had an excellent memory, doubtless assisted by his method of reading, which was a model of excellence. Every thing he examined and weighed critically and accurately, noted what was worthy of being remembered, and rose from the perusal of a book perfectly qualified to express an opinion of its merits, and to give a history of its contents. He never permitted himself, or those subject to his advice, to consume valuable time, and acquire vicious habits of reading, in the perusal of unworthy productions. His poetical taste and knowledge of classical English and foreign poetry were highly cultivated. Gœthe and Schiller, in their own vernacular, were his favorite authors. There was a period, as I have already mentioned, in his early history, when he himself had poetical inspirations, of which the proofs remain in two M. S. volumes. Although he particularly requested that these might remain private, there is no impropriety in mentioning that the volume examined by me contains several creditable effusions, of which the master piece is a fine poem on memory, occupying many pages.

In the ordinary affairs of life, his opinions, though occasionally impulsive, were generally correct, and always disinterested; few have been more chivalrous in asserting and maintaining the truth and justice of their convictions. In him, the rare sense of rectitude, maintaining nothing that is not scrupulously correct and true, *honesty of purpose*, was a noble trait. He held no communion with policy, he advocated no proceeding that could not repose on its own merits. In the examination of medical students, no persuasion could induce him to reconsider an unfavorable vote, conscientiously and advisedly given against one whom he deemed unworthy to be a doctor of medicine.

I have already said that he had a heart formed for friendship. What others bend to private motives was, in him, the indulgence of a noble impulse. Refined and cultivated in his tastes, instructed in every department of literature and science, copious and emphatic in diction, and of a sprightly and convivial temper, he brought to society every requisite for its enjoyment, and every title to its distinctions. The confidence which he exhibited in his own opinion and judgment as a physician, inspired a similar feeling in others; the kind and unaffected interest which he frequently displayed in the welfare of his patients, was the source of many grateful demonstrations. His reputation as an intelligent and scientific practioner was not limited to the immediate circle of his own practice; no one enjoyed in a higher degree the confidence and esteem of men of his own profession, and none have been more often called in consultation to render valuable aid in obscure, difficult, and dangerous cases.

The writings of Dr. Harrison are voluminous. Those published consist of his Essay towards a correct theory of the nervous system, containing, also, sundry appendices and notes; of three papers on the yellow fever, one on nutrition, one on the vital principle, one on the coagulation of the blood, an introductory lecture, and various reviews, notices, and translations, all published at different periods in the New Orleans Medical and Surgical Journal. Among his manuscripts, it is impossible to fix the order of their composition. We have private journals, case books, various volumes of excerpta, two of poems, and a large work on physiology, under the title of Vital Dynamics. To the latter properly belongs an extensive essay on sensation, occupying about ninety pages. The notes of his lectures are very brief.

Until his association with the New Orleans Medical and Surgical Journal, it is well known that he had paid comparatively little attention to medical literature. He had good reason, however, for a change of views since that period; a critical examination of the periodical publications and new works had brought up his knowledge to the most advanced state of science, and the favorable reception of his essays on yellow fever, and other efforts in this new field, encouraged a perseverance, which, with the continuation of his valuable life, would doubtless have been productive of important results.

The members of this society are all too familiar with the printed works of our late fellow, to require an extended analysis of their contents. They will, therefore, be briefly noticed in their chronological order, to fulfil the expression of the will of the society, and in respectful memory of their deceased author.

The essay on the Nervous System, the work by which the literary and scientific reputation was first established, has been so generally and extensively noticed in American and Foreign Journals, that, in my opinion, a synopsis of several will, under present circumstances, subserve the immediate purpose in view, and offer, at the same time, a proper idea of the estimation in which this production has been held by those whose pursuits and scientific character gave them best opportunity and right to express an opinion.

The publication of the Essay towards a correct theory of the nervous system elicited a copious bibliographical notice in the April number of the American Journal of the Medical Sciences for 1844. The able reviewer has done justice to the importance of the subjects adduced, and to the power with which the author's positions are sustained. The leading views of the relation of life to organization, and of the reference of vital actions to chemical changes, are faithfully expounded and well illustrated by frequent extracts from the work. Addressing himself to readers, he says: "It is only from an attentive perusal of the essay, that they can acquire a knowledge of the manner in which these views are sustained and elucidated by a reference to the established laws and phenomena of purely chemical action, as it occurs between different kinds of inorganic matter. The analysis of the application of the new chemical doctrines of insomerism and catalysis, as applied by Dr. Harrison, to explain the obscure phenomena of nervous action, gives a very correct idea of his favorite theories, and thus concludes: "We do not profess, ourselves, to be converted to all the doctrines which the author has advanced, and think that we are able to perceive more than one hiatus in his chain of reasoning. We nevertheless freely admit that, as a whole, his theory of the nervous system is one more consistent with what we know of the vital actions of the animal organism, and attended with mnch fewer difficulties, than any of those previously advanced; while in its exposition and defence, the author has exhibited not a little ingenuity, with a very thorough acquaintance with the present state of physiological and chemical knowledge, from the prominent facts of which he has very skilfully chosen the material for the support of his theory." So much for the views of one of the most scientific and intelligent collaborators of our first American Journal.

We have another, and a briefer notice, in Forbes' British and Foreign Medical Review for October, 1844. Alluding to the application of isomerism, already mentioned, to the explanation of nervous phenomena, Dr. Forbes proceeds: "This idea is expanded by Dr. Harrison with considerable acuteness and sagacity. He is evidently a man of great powers of reasoning and of observation, and we are disposed to consider many of his views as much more philosophical than speculations of this kind usually are. The author's clearness of thought and expression are well displayed in his examination of the electric theory of nervous action, and in his disquisition on life, which are contained in the appendix."

In addition to these notices, others more favorable appeared in the Edinburgh and Dublin Journals, which it has not been in my power to procure in time for this paper. One was also published in a French Journal, translated into German, and sent to the author, containing a very long and favorable analytic review of the whole work.

Enough has been said, at this time, to present the judgment pronounced by men of science on the learning and ability exhibited by the author of the work in question. Five years have elapsed since its publication, yet we believe that none of the novel views suggested have been adopted or sustained in the systematic treatises on physiology. The same, however, cannot be averred of the grounds that he has taken in opposition to the existence of a vital principle. The 2d appendix on life, occupying about thirty-two pages, is an admirable specimen of logical analysis, admired by all capable of appreciating its merits. So profoundly was he impressed with the importance of this subject, that he republished this appendix in the Medical Journal for July, 1846, fortified by an interesting extract from Mulder.

There is an additional note in the Essay, on the vitality of the blood. This subject was also continued in a "review of opinions, concerning the cause of the coagulation of the blood." In this, he recurs to the explanation of this phenomenon by the advocates for the influence of the vital principle, and attempts to show that it is a result of chemical action. Although referring no longer to the doctrines of insomerism and catalytic action, which formed the salient points of the Essay, he advocates similar views in relation to the rationale of the solidification of the fluid fibrine, by citing analogous examples offered by cyanic acid, chloral and aldehyde, which become solid without a change of their elements. A few years more, and the vital principle will be entirely discarded from the forces acknowledged by physiology and pathology. It is somewhat doubtful, however, whether the substitution of chemical action to explain all the phenomena of life is not somewhat premature, and whether, tested by the same severe logic, such explanations would be much more satisfactory or more incontrovertible than those we have abandoned. In September, 1845, Dr. Harrison commenced, in the New Orleans Medical and Surgical Journal, his remarks on yellow fever. After stating that he will confine himself to his own convictions and experience, without reference to the writings and opinions of others, and after indicating the opportunities he has enjoyed for observation, he writes: "I make these remarks for the purpose of showing what opportunities I have enjoyed of studying the disease, and if, in the present paper, there be found little that is new, or of much value in a positive sense, it may still, I hope, be of some service in destroying erroneous notions of the disease, contracted by those who have never seen it."

I am confident of being sustained by the experience of this society, in declaring, that notwithstanding the limited character of these remarks on yellow fever, they are far superior to any thing that has been published on that subject, and do infinite credit to our medical literature. Few could have been selected so well qualified in every respect to accomplish the task undertaken.

The description of symptoms given is accurate, methodically digested, and better calculated to impress, upon those desirous of acquiring information, a distinct idea of the different stages and forms of this remarkable disease than any thing we have seen. The post mortem appearances are such as could only be presented by one intimately acquainted with lesions of structure, and enjoying unlimited opportunities for observing what he attempts to describe. The pathology of yellow fever, particularly as regards the modus operandi of the supposed poison upon the circulation and general system; the rationale of lesions; the connection of morbid changes with symptoms; the nature of the passive congestions and hemorrhages, and the presumptive origin and composition of black vomit have received every thing from his hands that the present state of medical science could demand. We think, however, that, in the analysis of the distinctive characters of symptomatic and idiopathic fevers, our friend has given in a premature adhesion to the hematological doctrines of Andral, and that it would be difficult to establish, in rheumatic fevers and all those associated with decided phlegmasia, either that the local condition precedes the general, or that the majority, if not all, of these are not preceded by changes in the circulation as interesting to the pathologist, though as obscure as those associated with the very disease in question.

The continuation of this subject in the Journal for November, 1845, commences with the prognosis, which is characterized by the same graphic and experienced aphorisms, for which the whole essay is distinguished. To those of us who have been accustomed, for many successive epidemics, to grapple with this frequent invader, the general remarks on the treatment appear to be particularly judicious. I refer to the necessity of immediate treatment, of good nursing, of quiet, of regimen; the horizontal position; equable temperature; ventilation, and, above all, of a constant recollection of the uncertain and treacherous nature of the malady.

In his description of the several modes of practice, Dr. Harrison has meted out full justice to the mercurial. With his objections to the depleting system, I cannot exactly agree. This practice, particularly as relates to general depletion, has lost ground with the profession, less from its injurious effects under judicious management, than from having ceased to be necessary.

Dr. Harrison's history of the introduction of sulphate of quinine in the treatment of the yellow fever of this city is one that will probably be considered as decisive. His description of its effects are accurate and unprejudiced, and has been well confirmed by subsequent experience. We will not detain the society with a subject so familiar. In concluding my examination of these parts of our author's remarks, I may say that he has not only faithfully given the results of his own experience, but those of the collective intelligence and observation of his medical brethren of this community.

The third of these essays was published in the same Journal for March, 1847, under the title of Speculations on the cause of Yellow Fever. Whereas the others contained only the opinions and experience of the author, this is one of his most elaborate productions, entering fully into the circumstances under which the so called poisonous influence is generated; limiting and defining its operation; examining minutely the whole subject of malarious emanations, organic putrefaction and its products; and quoting largely the experiments of Gaspard, Magendie, Liebig, and others, and the observations of the most respectable authorities on the diseases of intertropical latitudes. The conclusions favor the local origin of the disease, influenced by meteorological conditions; disprove its mere miasmatic derivation; oppose its communicability, and advocate the existence of a substance which, either in the form of a volatile oil or other organic matter, held in solution by ammonia, floats in the atmosphere, is inhaled during the respiratory movements, taken into the circulation, and poisons the system. The hygienic suggestions for the eradication of this endemic from our city are such as every scientific advocate of its prosperity will readily approve. If, in the opinion of this society, it is expedient to republish the writings of our deceased fellow, I would recommend these observations on yellow fever to be the first selected for that object. Their real merit, and the just celebrity they have already acquired, will insure success to so laudable an experiment, equally creditable to the author and to the object.

A paper on the "nutritive process, tending to show that it is one of the forces of the circulation," was read before this society on February 7th, 1846, and published by its request in the May number of the New Orleans

Medical and Surgical Journal for the same year. The society having heard this essay, and expressed an opinion on it, will render it useless for me to examine it on this occasion.

I have already suggested to this society the propriety of erecting a monument to the memory of a fellow who has done more to elevate the medical literature and medical character of this State, than any other member of the medical profession. Let it be done under the authority of this body, and opened to public contribution—that we may once behold a generous and honorable distinction conferred on a member of the medical profession. Truth, ability, genius, and learning, are not common attributes. They are the candidates for that glorious immortality that philosophy has foreshadowed, and divinity revealed. We should cherish them while living. We should bear enduring and honorable testimony to their memory. These were all combined in John Harrison.—*N. O. Med. and Surg. Journal.*

